# Ten years of gadolinium retention and deposition: ESMRMB-GREC looks backward and forward

**DOI:** 10.1007/s00330-023-10281-3

**Published:** 2023-10-07

**Authors:** Aart J. van der Molen, Carlo C. Quattrocchi, Carlo A. Mallio, Ilona A. Dekkers, for the European Society of Magnetic Resonance in Medicine, Biology Gadolinium Research, Educational Committee (ESMRMB-GREC)

**Affiliations:** 1 Department of Radiology, C-2S, Leiden University Medical Center, Albinusdreef 2, NL-2333 ZA Leiden, The Netherlands; 2 Centre for Medical Sciences CISMed, University of Trento, 38122 Trento, Italy; 3 Department of Medicine and Surgery, Università Campus Bio-Medico di Roma, Roma, Italy; 4 Operative Research Unit of Diagnostic Imaging, Fondazione Policlinico Universitario Campus Bio-Medico, Roma, Italy

**Keywords:** Contrast media, Gadolinium, Magnetic resonance imaging, Brain, Body

## Abstract

**Abstract:**

In 2014, for the first time, visible hyperintensities on unenhanced T1-weighted images in the nucleus dentatus and globus pallidus of the brain were associated with previous Gadolinium-based contrast agent (GBCA) injections and gadolinium deposition in patients with normal renal function. This led to a frenzy of retrospective studies with varying methodologies that the European Society of Magnetic Resonance in Medicine and Biology Gadolinium Research and Educational Committee (ESMRMB-GREC) summarised in 2019. Now, after 10 years, the members of the ESMRMB-GREC look backward and forward and review the current state of knowledge of gadolinium retention and deposition.

**Clinical relevance statement:**

Gadolinium deposition is associated with the use of linear GBCA but no clinical symptoms have been associated with gadolinium deposition.

**Key Points:**

*• Traces of Gadolinium-based contrast agent-derived gadolinium can be retained in multiple organs for a prolonged time.*

*• Gadolinium deposition is associated with the use of linear Gadolinium-based contrast agents.*

*• No clinical symptoms have been associated with gadolinium deposition.*

**Supplementary Information:**

The online version contains supplementary material available at 10.1007/s00330-023-10281-3.

## Introduction

Gadolinium-based contrast agents (GBCA) are routinely used in patients undergoing magnetic resonance imaging (MRI) to enhance image contrast and thereby improve the detection and characterisation of lesions. Since their introduction in 1988, an estimated 750 million doses have been delivered and the current estimated use is 59 million doses per year (Bayer AG estimates based on various internal and external data, 2023 [1–3]). Overall, 30–45% of the MRI scans have used GBCA, with high contribution by Neuroradiology (~40%) and Cardiovascular Radiology (~20%) (Bayer AG estimates, based on various internal and external data, 2023).

Gadolinium deposition in the brain was first described in 2014. It was suggested that the retrospectively observed hyperintensity of the dentate nucleus (DN) and the globus pallidus (GP) relative to the pons (i.e., dentate nucleus to pons (DNP) ratio) on unenhanced T1-weighted (T1w) images of a population of patients with brain tumours was related to repeated administrations of linear GBCA [4]. Almost simultaneously, a European group reported similar findings on unenhanced T1w brain images after multiple injections of gadodiamide in patients with multiple sclerosis and in patients with brain metastases [5].

Of interest, a study on multiple sclerosis (MS) patients in 2009 already reported on hyperintensity of the DN on unenhanced T1w images in 23/119 patients. All patients had clinical symptoms of secondary progressive MS, and at the time the study did not associate this finding with previous contrast-enhanced MRI with linear GBCA [6].

### GBCA basics

A basic understanding of GBCA physicochemistry, transmetallation, and elimination is needed for understanding gadolinium deposition. For more detailed information see the Online Supplement to this article.

GBCA exploits the highly paramagnetic gadolinium (Gd), which shortens T1 and T2 of tissues, leading to increased signal intensity (SI) on T1w images (and reduced SI on T2-weighted (T2w) images).

Gadolinium (Z = 64 and MW = 157.25 g/mol) is an element from the Lanthanide family of elements that has the largest possible total spin (S = 7/2), and consequently a large spin magnetic moment [7]. The efficiency of T1w contrast agents in aqueous solutions is determined by their relaxivity r_1_ (r_1_ · [C] = 1 / ΔT_1_), which depends on temperature, field strength, and type of solution.

Unchelated Gd^3+^ ions are toxic because the ion has an ionic radius (107.8 pm) close to the ionic radius of Ca^2+^ (114 pm) and can bind to Ca^2+^ ion channels and Ca^2+^-dependent proteins such as metalloenzymes or messenger proteins like calmodulin or calexcitin. To avoid this potential toxicity, the Gd^3+^ ions must be tightly bound as a chelate. In Europe, such ligands have a macrocyclic (DOTA in gadoterate, BT-DO3A in gadobutrol, HP-DO3A in gadoteridol) or linear (BOPTA in gadobenate; EOB-DTPA in gadoxetate) structure.

The stability of the gadolinium-ligand complex can be described by several constants. The thermodynamic stability constant K_therm_ describes the affinity of Gd for the ligand at pH = 14.

For biological systems, the conditional thermodynamic stability constant K_cond_ is more appropriate. This characterises the affinity of gadolinium for ligands in aqueous media under physiologic conditions (pH = 7.4). The kinetic stability describes the kinetic rate of the dissociation of the ML complex under acidic conditions at pH = 1. The kinetic stability is in vivo the most important stability parameter [8].

Transmetallation is the exchange between Gd^3+^ and other metal ions M^+^ and depends on the stability of the chelating ligand. Gd^3+^ ions can be removed from the chelate by several ions like Zn^2+^, Cu^2+^, and Ca^2+^. When Gd^3+^ is released, it can form insoluble toxic Gd^3+^ compounds like GdPO_4_ or Gd_2_(CO_3_)_3_ [8].

After intravenous administration, extracellular GBCA is excreted by the kidneys with an early elimination half-life < 2h in patients with normal renal function, while > 95% of the GBCA is cleared from the body within 12 hours; both are similar for linear and macrocyclic GBCA. Hepatobiliary GBCA has additional intracellular transient uptake and hepatic excretion into the biliary tree. In patients with severely reduced renal function (estimated glomerular filtration rate (eGFR) < 30 ml/min/1.73 m^2^) this early elimination half-life can increase up to 30h [9], which can increase the likelihood of transmetallation. A review of pharmacokinetic data showed the presence of a deep compartment of distribution with long-lasting residual excretion. So far, the exact components of this deep compartment are unknown. This long-lasting excretion is faster for macrocyclic GBCA and is correlated to the higher thermodynamic stability and differences in transmetallation [10].

### Gadolinium deposition in the brain

#### Extracellular linear GBCA

Preclinical studies in rat brains have highlighted the occurrence of in vivo dechelation of Gd^3+^ ions from less stable GBCA, regardless of the presence of renal dysfunction and with a clear dose-effect relationship. All quantities were in the nmol /gram dry tissue range. It has also been shown that differences exist in the amount of total gadolinium retained in the brain between different GBCA compounds [11–14].

**Fig. 1:**
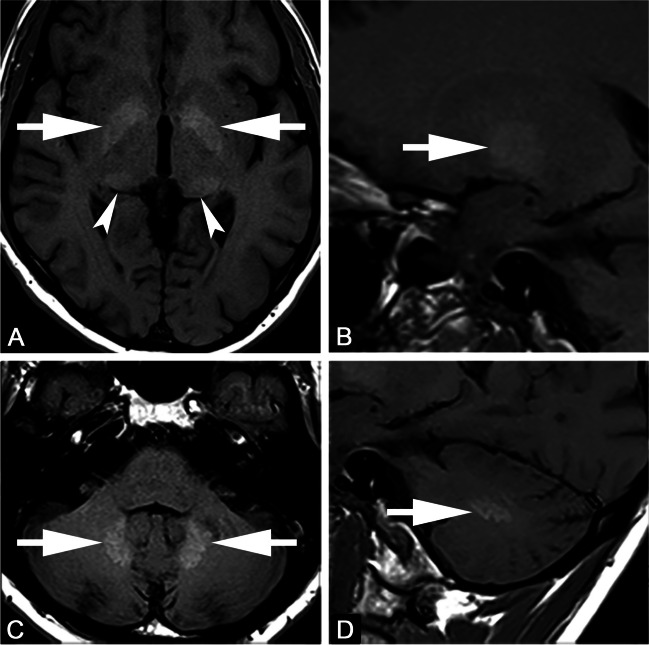
Axial (**A**, **C**) and sagittal (**B**, **D**) T1-weighted images. Forty-seven-year-old male with multiple sclerosis previously exposed to 14 intra-venous injections of gadodiamide. Globus pallidus and dentate nucleus hyperintensity on unenhanced T1-weighted images due to gadolinium deposition (arrows in **A**–**D**). Faint T1 hyperintensity of the pulvinar thalami is also seen (arrowheads in **A**)

**Fig. 2:**
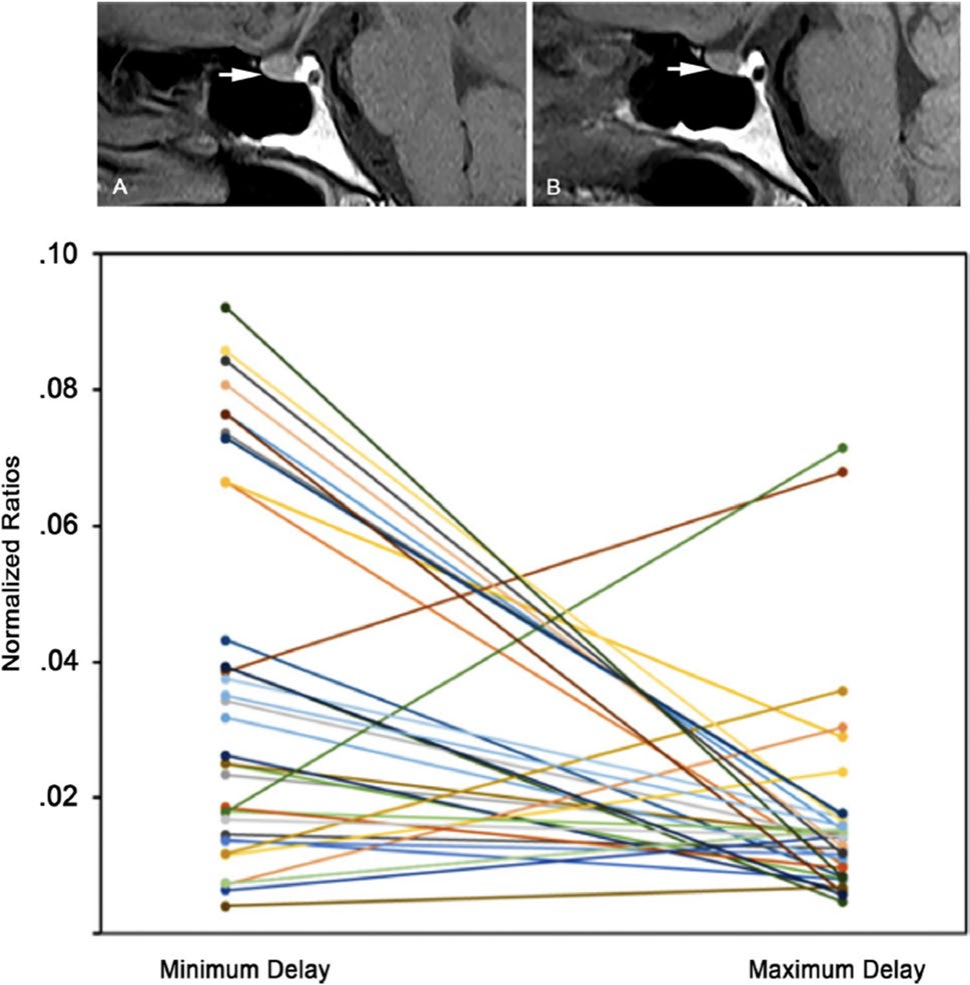
Slightly greater T1 signal intensity of the anterior pituitary gland in sagittal T1W image acquired at post-injection time delay of 1 day and only 1 previous GBCA exposure (arrow in **A**) with respect to a similar image acquired at post-injection time delay of 180 days and four previous GBCA injections (arrow in **B**). The lower panel shows the decreasing trend of normalised ratios from minimum to maximum post-injection time delay of each individual patient. Reproduced from reference 24 under the Creative Commons Attribution 4.0 International License (http://creativecommons.org/licenses/by/4.0/)

The use of linear extracellular GBCA led to visible changes in SI ratios and measurable Gd depositions in the rat and dog brains [15–18]. Most depositions were in perivascular foci in the DN and GP [15], with evidence of co-localisation to parenchymal iron [18]. The amount of deposition in rat brains occurred independent of age or sex [17]. Local blood-brain barrier disruptions did not lead to an increase in T1 SI ratios or Gd deposition [19]. Active inflammation showed higher Gd concentration in inflamed areas in mouse brains [20], while the presence of diabetes led to lower brain concentrations [21]. There was a decreased concentration over time in all brain regions, but long-term retention over 1 year occurred preferentially in the rat DN [16]. Despite DN and GP being the brain structures mostly involved by Gd deposition (Figure 1), it should be mentioned that Gd was also suggested to be deposited in other brain areas including pulvinar thalami, pons, frontal lobe cortex and white matter, and cerebellar cortex and white matter, mainly at the level of capillary endothelium and neural interstitium [18, 22, 23]. In addition, increased T1 signal intensity of the anterior pituitary gland, notably not lined by blood–brain barrier, has also been reported after serial exposure to extracellular linear GBCA (Figure 2) [24, 25].

Preclinical long-term studies have shown that for linear GBCA a large portion of gadolinium was retained in the brain, with binding of soluble Gd to macromolecules. For macrocyclic GBCA only traces of the intact chelated gadolinium were consistently reported, with complete washout over time [26, 27].

After the initial reports, a plethora of small retrospective studies was published. In the ESMRMB Gadolinium Research & Education Committee (ESMRMB-GREC) systematic review of these, increased SI in the dentate nucleus and or globus pallidus was found for linear GBCA, but no increases for macrocyclic GBCA, even after large doses [28].

The ESMRMB-GREC systematic review [28], but also a review of animal studies [29], showed that there was a large variety in sequence types and evaluation methodologies. The main problem is that increased SI ratios at unenhanced T1w MRI are a poor biomarker for gadolinium deposition, as SI ratios do not have a linear relationship with Gd concentration and are highly dependent on the MRI parameters used during acquisition and tissue gadolinium speciation. Absolute signal intensity (in arbitrary units) in MRI depends on many MRI parameters such as field strength, sequence type/parameters, coil sensitivity/filling factor, coil tuning/matching drift, etc. Signal intensities, or changes thereof, do not reflect true changes in Gd content [3, 28].

Intact GBCA doesn’t cross the intact blood-brain barrier. It is now believed that GBCA can reach the cerebrospinal fluid (CSF) via the choroid plexus and ciliary body and can reach the brain interstitium via the glymphatic system along perineural sheaths and perivascular spaces of penetrating cortical arteries [30]. It has been pointed out that all GBCA enter the cerebrospinal fluid glymphatic pathway through these anatomical structures not lined by the blood-brain barrier, then, transmetallation with other metals, such as Zn^2+^, Cu^2+^, and Fe^3+^, might take place at the extra-vascular space thanks to the high affinity for gadolinium-binding chelators [31, 32]. The reason for preferential Gd retention in particular brain nuclei or regions is unknown and remains an intriguing question. It might depend upon poorly understood anatomical peculiarity, vulnerability, or molecular frameworks occurring at specific brain structures. Indeed, the glymphatic system may serve as a pathway for Gd dechelated from linear GBCA but also serves as a physiological clearance route of more stable GBCA [31, 32].

GBCA distributed into the CSF cavity via the glymphatic system may remain in the eye or brain tissue for a longer duration compared to GBCA in systemic circulation. The potential retention of GBCA in the aqueous humor of the anterior chamber has been observed in children due to post-injection leakage [33]. Another paper from the same group reported signal intensity increase in various cerebral fluid spaces, including the vitreous body of the eye, on GBCA-enhanced T2w images, obtained at three hours post-injection in neurologically healthy adults [31].

#### Hepatobiliary linear GBCA

The use of gadobenate and gadoxetate has been restricted by the European Medicines Agency (EMA) to hepatobiliary MRI indications. The approved dose of gadoxetate is 0.025 mmol/kg and of gadobenate 0.05 mmol/kg, less than the dose of linear extracellular GBCA. However, outside the EU gadobenate is used for body MRI indications in doses up to 0.1 mmol/kg.

In sheep, the level of Gd retention 10 weeks after a single dose injection was 14-fold higher for gadobenate than for gadoterate [34]. In humans, the use of gadobenate led to visible SI changes in the brain [35, 36]. Neuroinflammation led to higher Gd concentrations in the rat brain after gadobenate use [37]. In human cadavers, the mean Gd concentration in the brain was 3–6 times higher for gadobenate compared to gadoterate. From time to GBCA administration to death, it was estimated that gadobenate washed out over time [38].

In an animal study after gadoxetate administration, no visible hyperintensity of the deep cerebral nuclei was demonstrated. Gadoxetate had lower cerebellar Gd levels than gadobutrol or gadodiamide [39]. Results of T1 hyperintensity in humans after gadoxetate administration were conflicting [40, 41], and a meta-analysis showed significant bias in five included studies in humans, and therefore available data on gadolinium deposition for gadoxetate is incomplete [42].

#### Macrocyclic GBCA

There was a consistent finding that cumulative dosing of macrocyclic GBCA did not lead to visible changes in SI on T1w images or changes in T1 relaxation times in rat and human brains [29, 32, 43, 44], but not in all studies [45].

In comparative studies in rats, macrocyclic GBCA led to measurable Gd concentrations at 1–5 weeks after administration, which were lower for gadoteridol compared to gadoterate and gadobutrol, independent of renal function [46]. The GBCA wash-out led to a 3–5-fold reduction from 1 to 5 weeks which was more rapid for gadoteridol. The levels at 5 weeks ranged from 0.14 to 0.30 nmol Gd/g tissue [47, 48].

#### R1 relaxometry and Quantitative Susceptibility Mapping (QSM)

MRI R1 relaxometry techniques [49] and QSM [50] are more sensitive tools for biometal imaging and allow the quantitative evaluation of transchelation of Gd from GBCA to competing macromolecules [51, 52]. After serial administration of gadobutrol relaxometry did not show R1 relaxometry changes in the DN [53, 54], but susceptibility changes on QSM could be demonstrated in the GP [52] or DN [55]. Brain radiotherapy can weaken the blood-brain barrier, which might lead to an increase in Gd accumulation with increased R1 relaxation in the DN [56]. Despite the exact contribution of radiotherapy remains somehow controversial, it is likely that brain irradiation is a co-factor enhancing the effect of GBCA on T1 signal intensity on unenhanced T1w images [57]. Indeed, in retrospective studies using SI ratios such as the ratio between globus pallidus and thalamus, the denominator can also be affected by gadolinium retention, and the choice of the reference region is thus crucial. Quantitative MRI approaches, such as R1 relaxometry or QSM can avoid this problem.

### Speciation of Gadolinium deposition in the brain

It is unclear what forms are responsible for the T1w signal increase. In the rat brain, three different chemical forms must be distinguished: intact GBCA, Gd bound to macromolecules (e.g., ferritin), and insoluble Gd-salts [58]. Intact GBCA was found for linear and macrocyclic GBCA, but the other forms were only for linear GBCA. As precipitated gadolinium does not induce any change in MRI signal, it is likely that the Gd bound to macromolecules is responsible for the visible T1w hyperintensity in clinical MRI [59].

In speciation analyses in rats exposed to intravenous gadobenate and gadodiamide, a combination of intact GBCA, complexes of dissociated Gd^3+^ bound to ferritin, and Gd^3+^ bound to other macromolecules was found. Incomplete column recovery suggested the presence of labile complexes of dissociated Gd^3+^ with other endogenous molecules. In addition, Gd was present in insoluble amorphous spheroid structures of 100–200 nm. Gd was consistently co-localised with calcium and phosphor, suggesting a composition of mixed Gd/Ca-phosphates [60, 61].

### Gadolinium deposition in the body

#### Abdominal organs

Most of the data regarding the abdominal organs is still largely investigational and no firm conclusions can be drawn yet.

In animal studies, residual Gd is also present in abdominal tissue samples [46, 62–66]. While deposition in the brain was only 2–7 μg Gd/g tissue, the amounts in other organs were much higher for kidney, liver, and spleen. The level was highest for gadodiamide [62]. In mice, high doses were found in the kidneys after high-dose gadodiamide (7.49 nmol/g tissue) or gadobutrol (16.36 nmol/g tissue) administration, but no spleen enlargement was found after GBCA administration [63]. In subtotally nephrectomised rats, higher Gd levels 28 days after administration of gadobutrol or gadoterate versus gadoteridol were determined in the kidneys and liver. After 56 days, lower Gd levels were determined for all GBCA [46].

In sheep, concentrations were 3–21 times higher for linear than for macrocyclic GBCA. Concentrations for kidney, liver, and spleen were for gadodiamide 879/780/137 ng/g, for gadobenate 179/157/16 ng/g, and for gadobutrol 86/35/6 ng/g tissue, respectively. No tissue alterations were detected [67]. In a study on rats, Gd was least retained after administration of gadoxetate, followed by gadobutrol and gadodiamide when clinically recommended doses were administered. Most of the retained Gd was excreted within 4 weeks after GBCA administration [68].

Administration of macrocyclic GBCA also led to measurable Gd concentrations in the liver and kidney 4 weeks after administration, which were lower for gadoteridol compared to gadoterate and gadobutrol. The levels for the liver ranged from 0.36 to 1.22 nmol Gd/g tissue and for the kidney 39–294 nmol Gd/g tissue [58].

**Fig. 3:**
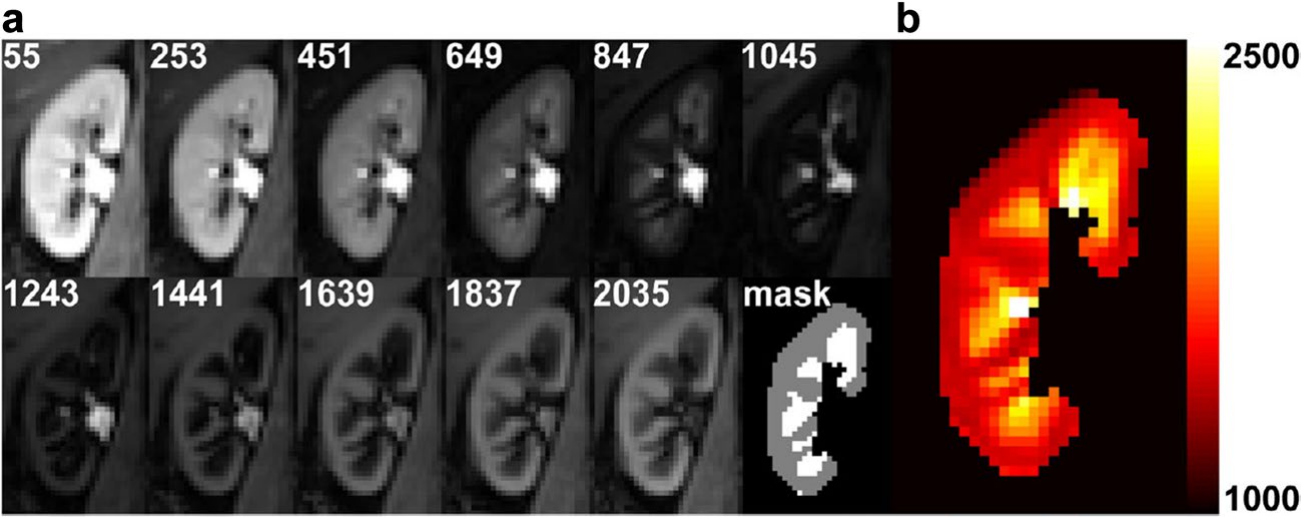
Example images of the right kidney from a healthy volunteer obtained at the first scan session. **a** T_1_ source image at multiple inversion times (in msec) after motion correction and the masks of the cortical and medullary segmentation. **b** Calculated corresponding T_1_ map. The color bar indicates T_1_ relaxation time in msec. The cortex and medulla can easily be discriminated thanks to the higher T_1_ in the medulla compared to the cortex. Reproduced from reference 69 under the Creative Commons Attribution 4.0 International License (http://creativecommons.org/licenses/by/4.0/)

**Fig. 4:**
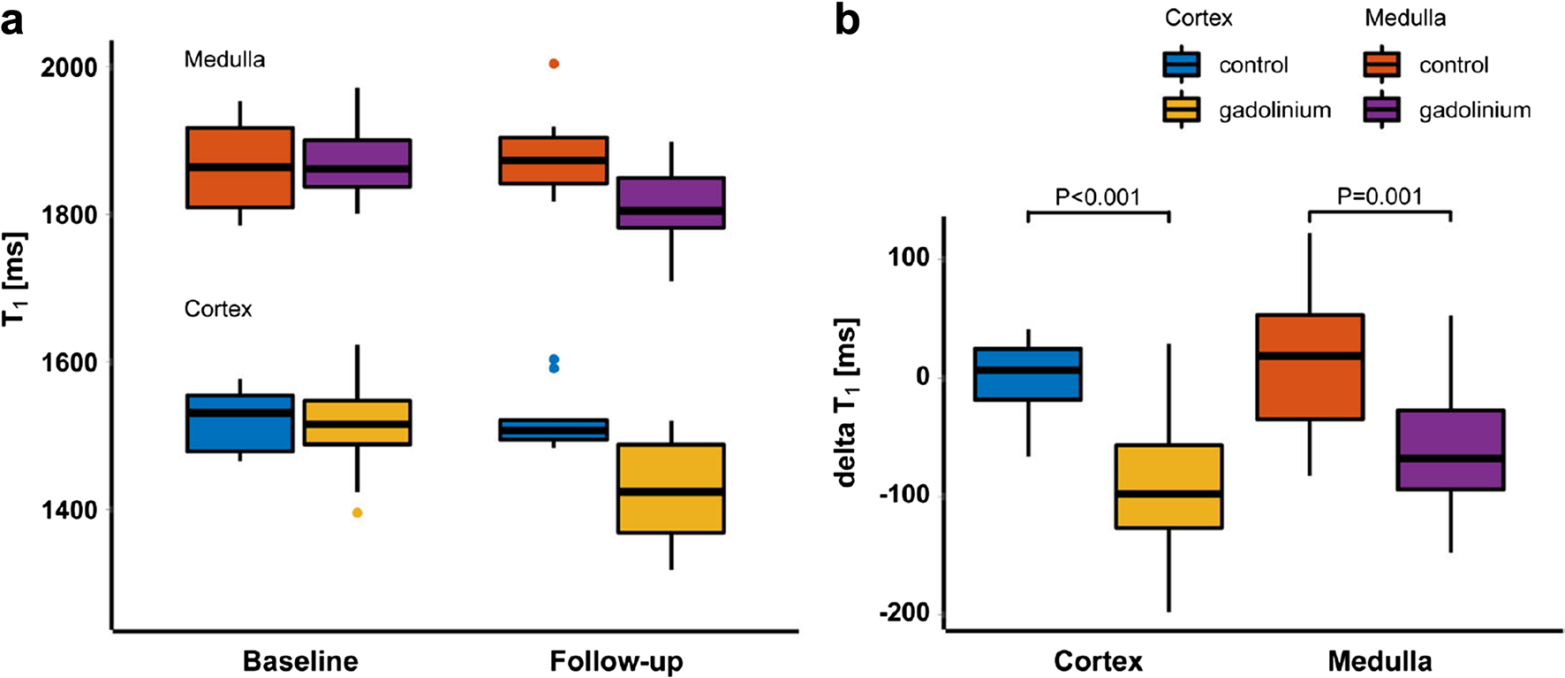
**a** Boxplots summarising the T_1_ values in the gadolinium (*n* = 16) and control group (*n* = 5) at baseline and follow-up. **b** Boxplots of the ΔT_1_ for cortex and medulla and both for the gadolinium and control group. ΔT_1_in the gadolinium group differed significantly from ΔT_1_ in the control group, both in the cortex (*p* < 0.001) and medulla (*p* = 0.001). Reproduced from reference 69 under the Creative Commons Attribution 4.0 International License (http://creativecommons.org/licenses/by/4.0/)

There is a paucity of data from human studies. Reduced T1 values in the renal cortex and medulla have been demonstrated after 7 days of a single dose of gadobutrol in subjects with normal renal function using T1 mapping [69]. This indicates the prolonged presence of small amounts of gadobutrol in the kidney after single-dose administration, suggesting delayed elimination of GBCA (Figures 3 and 4).

Gd deposits have been associated with iron overload in the livers of pediatric stem cell transplantation patients with normal renal function [70].

### Bone

Lanthanide metals (gadolinium, samarium, europium, and cerium) have long been known to deposit in bone tissues and have effects on osteoblasts and osteoclasts, although the exact mechanisms are not well understood [71]. Gadolinium deposits have been found in samples of bone tissues of humans at higher concentrations than in brain tissue after administration of linear and macrocyclic GBCA, whereby linear GBCA deposits 4 to 25 times more than macrocyclic GBCA [66, 72, 73].

Bone residence time for macrocyclic GBCA (up to 30 days) is much shorter than for linear GBCA (up to 8 years) [10, 72]. The bone may serve as a storage compartment from which Gd is later released in the body [74]. It is postulated that the long-term reservoir of gadolinium in bones might implicate that some patients with high bone turnover (post-menopausal women, osteoporosis) may be more vulnerable to gadolinium deposition in bone [72].

Again, human data is scarce. In a cadaver study, 80 days after last GBCA exposure the mean Gd concentration in bone and skin was 2.9–4.4 times higher for gadobenate compared to gadoterate. Bone was the primary Gd retention site with levels of 23–100 ng/g tissue/mmol GBCA, while the Gd elimination rate was high for skin [38].

#### Skin

Gadolinium deposition in the skin has been demonstrated ever since the association of GBCA with nephrogenic systemic fibrosis (NSF) in 2006. In rat skin, administration of macrocyclic GBCA led to measurable Gd concentrations 1–5 weeks after administration, which were lower for gadoteridol compared to gadoterate and gadobutrol. The levels in the skin were initially high, but after washout levels at 5 weeks ranged from 0.31 to 0.53 nmol Gd/g tissue [47, 48].

In skin biopsies of NSF patients, gadolinium was found along collagen bundles but also as insoluble apatite-like deposits, suggesting dechelation [75, 76]. After linear GBCA, gadolinium deposits were found up to 40–180 times more frequently than after macrocyclic GBCA, histologic changes are more extensive, and products of dechelation of GBCA can be found [66, 77]. Gd is also deposited in the skin of patients with normal renal function after high cumulative GBCA doses [78]. With normal renal function ‘gadolinium-associated plaques’ have been described after gadodiamide, suggesting that Gd deposition in the skin after linear GBCA might give clinically relevant symptoms [79].

### Possible clinical symptoms of gadolinium deposition

Despite the retention or even deposition of Gd in various tissues, no histopathologic changes in rat brains could be found [80], nor tissue alterations in MS patients [81]. In addition, no effect on sensorimotor or behavioural functions could be demonstrated for either linear or macrocyclic GBCA in mice [82] or in humans [83]. Gadolinium retention was not related to symptom worsening in relapsing MS patients [84, 85] nor to Parkinsonism [86].

For linear GBCA, pain hypersensitivity has been seen in rats [87]. In MS, increased relaxation rates may be associated with lower information-processing speed [43] or mild effects on cerebellar speech or verbal fluency [43, 81], but these couldn’t be fully attributed to GBCA. Dermal thickening of the scalp skin has been reported in MS patients with normal renal function exposed to linear GBCA as compared to a matched group of patients exposed to macrocyclic GBCA, thus suggesting subclinical chronic effects of gadolinium retention on the skin [88].

### The European Medicines Agency ruling

The described association between NSF and exposure to linear GBCA in 2006 resulted in a switch to macrocyclic GBCA only (mostly gadoterate or gadobutrol) use in many European hospitals from 2007 onwards.

After publications describing increased signal intensities in the brain nuclei on unenhanced T1-weighted imaging after multiple linear GBCA exposures and post-mortem studies revealing the presence of small amounts of gadolinium in neural tissues, the EMA instituted a pharmacovigilance referral procedure (article 31). This led to the withdrawal of EU market authorisations of the high-risk linear GBCA gadodiamide and gadoversetamide and restricted the use of gadopentetate to MR arthrography and gadobenate to liver MRI [89, 90]. In Europe, only macrocyclic GBCA are available for general use, while gadoxetate and gadobenate are available for liver MRI (Table 1).

**Table 1:** Reviewed studies describing findings of gadolinium retention/deposition for various GBCA, stratified by involved organs

Contrast agent	Organs involved	Major findings	References
*Linear GBCA*			
Gadodiamide	Brain, pituitary gland	Hyperintensity in DN and GP on unenhanced T1w (humans)	4,5
		Hyperintensity in DN and GP on unenhanced T1w (animals)	11,12,13,14,15,16,17,35,68
		No ultrastructural or metabolic changes (animals)	14,15,16,17
		Hyperintensity in anterior pituitary gland on unenhanced T1w (humans)	24,25
		Persistent hyperintensity in DCN on T1w after 12 months (animals)	26,27
		25-40% washout of brain within 12 months (animals)	26,27
		Increased R1 relaxation rate (humans)	44
		No increase in T1 hyperintensity after radiotherapy (animals)	58
		Gd present as intact chelate, soluble macromolecules, insoluble forms (animals)	59,60
		Higher SI changes in DN on unenhanced T1w than macrocyclic GBCA (animals)	63
	Liver, spleen, kidneys	Higher Gd levels in liver, spleen, kidneys than macrocyclic GBCA (animals)	63,67,69
		Higher level of Gd in spleen than gadobutrol (animals)	64
		More kidney fibrosis, amyloid, vasocongestion than gadoterate (animals)	65
		High Gd levels in liver and kidney (animals)	68
	Skin, bone	Very long bone residence time (humans)	10
		Higher level Gd in skin and bone than gadoterate (animals)	67,76
		Higher Gd levels in femoral bone than gadoteridol and controls (humans)	73,74
		Dermal thickening in multiple sclerosis patients	89
Gadopentetate	Brain	Not significant hyperintensity in DN and GP on unenhanced T1w (animals)	11
		Hyperintensity in DN and GP on unenhanced T1w (animals)	13
		10-40% washout of brain within 12 months (animals)	27
		Hyperintensity in DN and GP on unenhanced T1w (humans)	36
		No increase in Gd levels after radiotherapy (animals)	19
		Increased Gd levels after brain inflammation (animals)	20
		Increased R1 relaxation rate (humans)	44
		Gd present as intact chelate, soluble macromolecules, insoluble forms (animals)	59
	Liver, spleen, kidneys	Higher Gd levels in liver, spleen, kidneys (animals)	66
	Skin, bone	Long bone residence time (humans)	10
Gadobenate	Brain	Hyperintensity in DN and GP on unenhanced T1w (animals)	11,13,35,63,68
		Hyperintensity in DN and GP on unenhanced T1w (humans)	36,37,39
		Increased Gd levels after abdominal sepsis (animals)	38
		Gd present as intact chelate, soluble macromolecules, insoluble forms (animals)	59
		Higher SI changes in DN on unenhanced T1w than macrocyclic GBCA (animals)	63
	Liver, spleen, kidneys	Higher Gd levels in liver, spleen, kidneys than macrocyclic GBCA	63,68
		High Gd levels in liver and kidney (animals)	68
	Bone, Skin	Intermediate bone residence time (humans)	10
		Higher Gd retention in bone than gadoteridol (humans)	39
		Intermediate Gd levels in skin (animals)	76
Gadoxetate	Brain	No hyperintensity in DN and GP on unenhanced T1w (animals)	40
		No hyperintensity in DN and GP on unenhanced T1w (humans)	41,43
		Hyperintensity in DN and GP on unenhanced T1w (humans)	42,43
	Liver, spleen, kidneys	Lower levels Gd in liver, spleen, kidneys than gadodiamide or gadobutrol (animals)	69
	Skin, bone	Intermediate bone residence time (humans)	10
*Macrocyclic GBCA*			
Gadoteridol	Brain	More than 65% washout of brain within 12 months (animals)	27
		No detectable Gd levels in brain (animals)	35
		Lower level Gd in brain than gadoterate and gadobutrol (animals)	47,48,49
		Gd only present as intact chelate (animals)	60
		Lower T1w SI changes in DN on unenhanced T1w than linear GBCA (animals)	63
	Liver, spleen, kidneys	Lower level Gd in kidney and/or liver than gadoterate and gadobutrol (animals)	47,48
		Lower Gd levels in liver, spleen, kidneys than linear GBCA (animals)	63
	Skin, bone	Lower level Gd in skin than gadoterate and gadobutrol (animals)	48,49
		Lower Gd levels in femoral head bone than gadodiamide (humans)	73,74
		Intermediate bone residence time (humans)	10
Gadoterate	Brain	No hyperintensity in DN and GP on unenhanced T1w (animals)	11,12,13,17
		More than 85% washout of brain within 12 months (animals)	26,27
		No detectable Gd levels in brain (animals)	35
		Higher level Gd in brain than gadoteridol (animals)	47,48,49
		Gd only present as intact chelate (animals)	59
	Liver, spleen, kidneys	Higher level Gd in kidney and/or liver than gadoteridol (animals)	47,48
		Less kidney fibrosis, amyloid, vasocongestion than gadodiamide (animals)	65
		Lower level Gd in liver than gadodiamide (animals)	67
	Skin, bone	Higher level Gd in skin than gadoteridol (animals)	48,49
		Lower level Gd in skin and bone than gadodiamide (animals)	67,76
		Short bone residence time (humans)	10
Gadobutrol	Brain	No hyperintensity in DN and GP on unenhanced T1w (animals)	11
		More than 85% washout of brain within 12 months (animals)	27
		No detectable Gd levels in brain (animals)	35
		Higher level Gd in brain than gadoteridol (animals)	47,48,49
		Higher magnetic susceptibility in GP (humans)	53
		Increased Gd accumulation by QSM after radiotherapy (humans)	57
		Gd only present as intact chelate (animals)	59
		Lower SI changes in DN on unenhanced T1w than linear GBCA (animals)	63
	Liver, spleen, kidneys	Higher level Gd in rat kidney and/or liver than gadoteridol (animals)	47,48
		Lower Gd levels in liver, spleen, kidneys than linear GBCA (animals)	63
		Lower level of Gd in spleen than gadodiamide (animals)	64
		Decreased renal T1 on unenhanced T1w (humans)	70
	Skin, bone	Short bone residence time (humans)	10
		Lower level Gd in skin than linear GBCA (animals)	11,76
		Higher level Gd in skin than gadoteridol (animals)	48,49

Abbreviations: *DCN* = Deep cerebral nuclei; *DN* = Dentate Nucleus; *GBCA* = Gadolinium-based contrast agent(s); *Gd* = gadolinium; *GP* = Globus Pallidus; *QSM* = Quantitative Susceptibility Mapping; *SI* = Signal intensity; *T1w* = T1-weighted

## Conclusions

After 10 years, even though there is evidence that GBCA are retained and that sometimes Gd is deposited in tissues, there is no evidence of clinical symptoms nor harm associated with Gd deposition in the brain and body. In practice, clinical radiologists ensure a strict indication for contrast-enhanced MRI and only use EMA-approved or American College of Radiology (ACR) grade II GBCA in all patients to minimise Gd deposition. But there are still many knowledge gaps about Gd metabolism and Gd deposition for which an international research agenda is important. We keenly await the results of ongoing studies that have been issued by the Federal Drug Authority on the joint contrast media manufacturers. In the meantime, the ACR/NIH/RSNA Agenda remains a good guidance document to target future research [3].

### Supplementary Information

Below is the link to the electronic supplementary material.

Supplementary file1 (PDF 209 KB)

## Abbreviations

ACR: American College of Radiology
CM: Contrast medium/media
DN: Dentate nucleus
DNP: Dentate nucleus-pons ratio
eGFR: Estimated glomerular filtration rate
EMA: European Medicines Agency
ESMRMB-GREC: European Society of Magnetic Resonance in Medicine & Biology Gadolinium Research & Educational Committee
GBCA: Gadolinium-based contrast agent/agents
Gd: Gadolinium
GP: Globus pallidus
L: Ligand
M: Metal
ML: Metal-ligand complex
MRI: Magnetic resonance imaging
MW: Molecular weight
NIH: National Institutes of Health
QSM: Quantitative susceptibility mapping
RSNA: Radiological Society of North America
SI: Signal intensity
T1w: T1-weighted
T2w: T2-weighted
Z: Atomic number

## Declarations

### Guarantor

The scientific guarantor of this publication is Aart J. van der Molen.

### Statistics and biometry

No complex statistical methods were necessary for this paper.

### Methodology

• retrospective

• multicentre study
