# Correction: Ten years of gadolinium retention and deposition: ESMRMB-GREC looks backward and forward

**DOI:** 10.1007/s00330-023-10446-0

**Published:** 2023-11-15

**Authors:** Aart J. van der Molen, Carlo C. Quattrocchi, Carlo A. Mallio, Ilona A. Dekkers, for the European Society of Magnetic Resonance in Medicine, Biology Gadolinium Research, Educational Committee (ESMRMB-GREC)

**Affiliations:** 1 Department of Radiology, C-2S, Leiden University Medical Center, Albinusdreef 2, NL-2333 ZA, Leiden, The Netherlands; 2 Centre for Medical Sciences CISMed, University of Trento, 38,122 Trento, Italy; 3 Department of Medicine and Surgery, Università Campus Bio-Medico di Roma, Roma, Italy; 4 Operative Research Unit of Diagnostic Imaging, Fondazione Policlinico Universitario Campus Bio-Medico, Roma, Italy

**European Radiology**

**https://doi.org/10.1007/s00330-023-10281-3**

In this article incorrect references are cited in the legends of Figs. 3 and 4:

Caption of Fig. 3 incorrecly states "Reproduced from reference 70 under the Creative Commons Attribution 4.0 International License" but it should have been "Reproduced from reference 69 under the Creative Commons Attribution 4.0 International License".

Caption of Fig. 4 incorrecly states "Reproduced from reference 70 under the Creative Commons Attribution 4.0 International License" but it should have been "Reproduced from reference 69 under the Creative Commons Attribution 4.0 International License".

The original article has been corrected.

