# Supplementary material for: Ten years of gadolinium retention and deposition: ESMRMB-GREC looks backward and forward
Source: Eur Radiol. 2023 Oct 7;34(1):600–11. doi: 10.1007/s00330-023-10281-3 (PMC10791848; doi:10.1007/s00330-023-10281-3)
Supplement: Supplementary file 1 — Supplementary file1 (PDF 209 KB) [file 330_2023_10281_MOESM1_ESM.pdf]

Ten years of gadolinium retention and deposition: ESMRMB-GREC looks backward and forward.

Electronic Supplementary Material

GBCA Physicochemistry

GBCA exploit the highly paramagnetic nature of gadolinium (Gd), which alters the local magnetic properties of protons by shortening T1 and T2 of tissues, leading to increased signal intensity on T1-weighted images (and reduced signal intensity on T2-weighted images).

Gadolinium (Z = 64 and MW = 157,25 g/mol) is a metallic rare earth element from the Lanthanide family of elements in the periodic system. Gd has the largest possible total spin (S = 7/2), and consequently a large spin magnetic moment [S1-S3].

The efficiency of T1-weighted contrast agents in aqueous solutions is determined by their relaxivity r_1 ($r_1 \cdot [C] = 1 / \Delta T_1$). The relaxivity depends on temperature, field strength, and type of solution. It is determined by relaxation effects of water molecules interacting directly with the paramagnetic Gd^{3+} ion (inner sphere) and interactions with closely diffusing water molecules without interacting with the metal-ligand (ML) complex (second or outer sphere). For clinically approved GBCA, about 60% of relaxivity comes from inner sphere and 40% from outer sphere effects. Chelated gadolinium coordination complexes are monohydrated ($Gd_{(H2O)}{}^{3+}$), as the ligands occupy 8 coordination sites and in their spherical configuration there is only enough space around the gadolinium for one (inner sphere) water molecule that exchanges rapidly with other nearby (outer sphere) water molecules [S4].

In biological systems, unchelated Gd^{3+} ions are toxic because the ion has an ionic radius (107,8 pm) close to the ionic radius of Ca^{2+} (114 pm) and can bind to Ca^{2+} ion channels and Ca^{2+}-dependent proteins such as metalloenzymes or messenger proteins like calmodulin or calexcitin. To avoid this potential toxicity, the Gd^{3+} ions

must be tightly bound as a metal-ligand (ML) complex or chelate. The ligand will reduce toxicity, change the tissue distribution, and influence relaxivity. Currently in Europe, such ligands have a macrocyclic (DOTA in gadoterate, BT-DO3A in gadobutrol, HP-DO3A in gadoteridol) or linear (BOPTA in gadobenate; EOB-DTPA in gadoxetate) structure.

Normally, equilibrium exists for the reaction between metal M and ligand L.
This reaction can be written as: (M) + (L) $\leftrightarrow$ (ML)

The stability of the gadolinium-ligand complex can be described by several constants. The logarithm of the thermodynamic stability constant K_{therm} describes the affinity of Gd for the ligand and is normally measured at pH = 14. Higher values imply a higher stability.
K_{therm} = (ML) / (M) · (L).

For biological systems the logarithm of the apparent or conditional thermodynamic stability constant K_{cond} is more appropriate. This is based on the total concentration of the free ligand, including all its protonation states, and it characterizes the affinity of gadolinium for ligand in aqueous media under physiologic conditions (pH = 7,4). In all GBCA the conditional stability is substantially lower than the thermodynamic stability.
K_{cond} = (ML) / (M) · {(L) + (HL) + (H_2L) + (H_3L) + ………}

The kinetic stability describes the kinetic rate of the dissociation of the ML complex. It is closely related to the thermodynamic stability and is commonly described as the half-life of the dissociation of the Gd-Ligand complex or by the observed dissociation constant k_{obs}. To be measurable, such kinetic analyses are done under acidic conditions at pH = 1 [S5].
Dissociation rate = k_{obs} (ML).

Some commercial solutions of contrast media contain variable amounts of free ligands or calcium complexes to ensure chelation of any free Gd^{3+} or other metal traces from the vial during its shelf life. The thermodynamic stability constants are a

measure of how much uncomplexed Gd^{3+} will be released in biologic tissues if the system reaches equilibrium. In vivo, such new thermodynamic equilibrium is usually not reached as most of the complex is excreted before any uncomplexed gadolinium can be released. Therefore, the kinetic stability is in vivo much more important than the thermodynamic stability.

Risk of transmetallation

Transmetallation is the exchange between Gd^{3+} and other metal ions M^+ that have greater affinity for the chelate. The amount of transmetallation depends on the stability of the chelating ligand. Gd^{3+} ions can be removed from the Gd-ligand (Gd-L) complex by several endogenous positively charged ions like Zn^{2+}, Cu^{2+}, and Ca^{2+}. When Gd^{3+} is released, endogenous negatively charged ions, like phosphate PO_4^{3-} and carbonate CO_3^{2-}, can compete with the free ligand to form insoluble toxic Gd^{3+} compounds like $GdPO_4$ or $Gd_2(CO_3)_3$ [S6].

Transmetallation can be described by: $(Gd\text{-}L) + (M^+) \leftrightarrow Gd^{3+} + (ML)$

In clinical imaging, a high kinetic stability of the metal-ligand complex is regarded as the most relevant stability parameter to minimize transmetallation. Since the stability of the macrocyclic Gd chelates is much more limited by the slow release of Gd^{3+} from the complex, the kinetic stability is more important in such ligands.

GBCA Biodistribution and Elimination

After intravenous administration, extracellular GBCA is excreted by the kidneys with an early elimination half-life of slightly less than 2h in patients with normal renal function. The hepatobiliary GBCA have additional intracellular transient uptake and hepatic excretion into the biliary tree. More than 95% of the injected GBCA is cleared from the body within 6 elimination half-lives, or 12h. This early excretion phase is similar for linear and macrocyclic GBCA. In patients with severely reduced renal function (estimated glomerular filtration rate (eGFR) < 30 ml/min/1.73m^2) the early elimination half-life can increase up to 30h [S7]. During this prolonged circulation, the

likelihood of transmetallation and release of free Gd^{3+} ions increase [S8].

A systematic review of pharmacokinetic data showed the presence of a deep compartment of distribution with long-lasting residual excretion. So far, the exact components of this deep compartment are unknown. This long-lasting excretion is faster for macrocyclic compared to linear GBCA and is correlated to the higher thermodynamic stability and differences in transmetallation. In addition, bone residence time for macrocyclic GBCA (up to 30 days) was much shorter than for linear GBCA (up to 2.5 years) [S9].

References

S1. Caravan P, Ellison J, McMurry TJ, Lauffer RB (1999) Gadolinium (III) chelates as MRI contrast agents: structure, dynamics, and applications. Chem Rev 99: 2293–2352.

S2. Hao D, Ai T, Goerner F, Hu X, Runge VM, Tweedle M (2012) MRI contrast agents: basic chemistry and safety. J Magn Reson Imaging 36: 1060–1071.

S3. Lin SP, Brown JJ (2007) MR contrast agents: physical and pharmacologic basics. J Magn Reson Imaging 25: 884–899.

S4. De León-Rodríguez LM, Martins AF, Pinho MC, Rofsky NM, Sherry AD (2015) Basic MR relaxation mechanisms and contrast agent design. J Magn Reson Imaging 42: 545-565.

S5. Port M, Idée JM, Medina C, Robic C, Sabatou M, Corot C (2008) Efficiency, thermodynamic and kinetic stability of marketed gadolinium chelates and their possible clinical consequences: a critical review. Biometals 21: 469-490.

S6. Idée JM, Port M, Raynal I, Schaefer M, Le Greneur S, Corot C (2006) Clinical and biological consequences of transmetallation induced by contrast agents for magnetic resonance imaging: a review. Fundam Clin Pharmacol 20: 563–576.

S7. Van der Molen AJ, Dekkers IA, Geenen RWF, et al; ESUR Contrast Media Safety Committee (2023) Waiting times between examinations with contrast media: a review of contrast media pharmacokinetics and updated ESUR Contrast Media Safety Committee guidelines. Eur Radiol, doi: 10.1007/s00330-023-10085-5.

S8. Aime S, Caravan P (2009) Biodistribution of gadolinium-based contrast agents, including gadolinium deposition. J Magn Reson Imaging 30: 1259-1267.

S9. Lancelot E (2016) Revisiting the pharmacokinetic profiles of gadolinium-based contrast agents. Invest Radiol 51: 691-700.
